# A novel delins (c.773_819+47delinsAA) mutation of the *PCCA* gene associated with neonatal-onset propionic acidemia: a case report

**DOI:** 10.1186/s12881-020-01102-1

**Published:** 2020-08-20

**Authors:** Hai-rong Wang, Yan-qiu Liu, Xue-lian He, Jun Sun, Fan-wei Zeng, Cheng-bin Yan, Hao Li, Shu-yang Gao, Yun Yang

**Affiliations:** 1 BGI-Anhui Clinical Laboratories, BGI-Shenzhen, Weisan Road, Fuyang, 236000 China; 2 Department of Genetics, Jiangxi Maternal and Child Health Hospital, No. 318 Bayi Road, Nanchang, 330006 China; 3 Department of Obstetrics and Gynecology, Wuhan Women and Children Medical Care Center, No. 100 Xianggang Road, Wuhan, 430015 China; 4 BGI Genomics, BGI-Shenzhen, Shenzhen, 518083 China; 5 Department of Obstetrics and Gynecology, The Second Affiliated Hospital of Zhengzhou University, Zhengzhou, 450014 China

**Keywords:** Propionic acidemia, Propionyl-CoA carboxylase, *PCCA*, Targeted NGS, Delins, BAM diagram

## Abstract

**Background:**

Propionic acidemia (PA)(OMIM#606054) is an inborn error of branched-chain amino acid metabolism, caused by defects in the propionyl-CoA carboxylase (PCC) enzyme which encoded by the *PCCA* and *PCCB* genes.

**Case presentation:**

Here we report a Chinese neonate diagnosed with suspected PA based on the clinical symptoms, gas chromatography-mass spectrometry (GC/MS), and brain imaging tests. Targeted next-generation sequencing (NGS) was performed on the proband. We detected only one heterozygous recurrent nonsense variant (c.937C > T, p.Arg313Ter) in the *PCCA* gene. When we manually checked the binary alignment map (BAM) diagram of *PCCA* gene, we found a heterozygous deletion chr13:100915039-100915132delinsAA (c.773_819 + 47delinsAA) (GRCh37.p13) inside the exon 10 in the *PCCA* gene. The results were validated by Sanger sequencing and qPCR method in the family: the variant (c.937C > T, p.Arg313Ter) was in the maternal allele, and the delins was in the paternal allele. When the mother was pregnant again, prenatal diagnosis was carried out through amniocentesis at 18 weeks gestation, the fetus carried neither of the two mutations. After birth, newborn screening was undertaken, the result was negative.

**Conclusions:**

We identified a recurrent c.937C > T and a novel c.773_819 + 47delinsAA mutations in the *PCCA* gene, which may be the genetic cause of the phenotype of this patient. Our findings expanded the spectrum of causative genotype-phenotype of the *PCCA* gene. For the cases, the NGS results revealed only a heterozygous mutation in autosomal recessive disease when the gene is associated with phenotypes, it is necessary to manually check the BAM diagram to improve the detection rate. Targeted NGS is an effective technique to detect the various genetic lesions responsible for the PA in one step. Genetic testing is essential for genetic counselling and prenatal diagnosis in the family to avoid birth defects.

## Background

Propionic acidemia (PA) (MIM #606054) is one of the classical inborn error of organic acid metabolism inherited in an autosomal recessive trait. Clinical manifestations of PA vary from neonatal-onset to late-onset forms. Neonatal-onset PA is the most common type which shows symptoms in the first few days after birth. The individuals usually manifest with poor feeding repeated vomiting, irritability, progressive encephalopathy, seizures, and lethargy [1]. With the progress of the disorder, the patient may experience a series of metabolic acidosis and complications influencing the neurologic, cardiologic, immunologic, hematologic, and gastrointestinal system. Without being treated appropriately in the acute period, it can lead to metabolic acidosis, coma, which can result in death. Patients with late-onset PA present with milder clinical symptoms [2], or undergo more multiorgan complications such as severe movement disorders and permanent neurologic damage [3], when suffering a metabolic crisis under catabolic stress. The prevalence of the disease varies in ethnic, with the highest reported 1:1000 in Greenlandic Inuits on account of a founder effect [4], 1:2000–1:5000 in some Saudi tribes due to consanguineous marriage [5], 1:105, 000–130,000 in the United States [6], and 1/85,000–1/186,000 in China [7, 8].

PA is caused by the deficiency of propionyl-CoA carboxylase (PCC) that catalyzes the conversion of propionyl-CoA to D-methylmalonyl-CoA. The disrupted function of PCC leads to abnormal mitochondrial accumulation of propionyl-CoA and its by-products. PCC, a mitochondrial biotin-dependent enzyme of ∼800 kDa, which is composed of α and β subunits in α6β6 form [9], encoded by the *PCCA* and *PCCB* genes, respectively. The *PCCA* gene is located on chromosome 13q32. It spans over 360 kb and contains 24 exons ranging from 37 to 335 bp in length, encoding a protein contains 728 amino acids (NM_000282). The *PCCB* gene is on chromosome 3q21-q22. It comprises 15 exons that encode a protein contains 539 amino acids (NM_000532).

There are several clinical reports of PA in Chinese population in recent years [10–14]. Although mutations are mainly found on the *PCCA* gene, no predominant mutations exist in Chinese PA patients [10].

Clinically, biochemical testing has been carried out for the screening and diagnosis of PA. Mass spectrometry (MS/MS) has been used in Mainland China since 2004, however, families must pay out of pocket for this test in the majority of regions [7]. The patient with PA shows increased propionyl carnitine (C3) in blood plasma by MS/MS. The result of the urine test by gas chromatography-mass spectrometry (GC/MS) shows elevated levels of methyl citrate, propionyl glycine, and 3-hydroxypropionate [15]. The initial diagnosis can be established based on the clinical manifestations and biochemical tests. However, clinical manifestations of PA are often nonspecific, especially in the infant period. In some cases, the patient showed no typical abnormal biochemical result, especially for the late-onset patient [12]. The combination of genetic analysis and biochemical tests may offer a more precise diagnosis Next-generation sequencing (NGS) has demonstrated the efficiency for the wide variety of variant classes, such as SNVs, INDELs, and structural variation for hereditary disorders [16, 17]. Here, we describe a case diagnosed with early-onset PA and identified the molecular defect by targeted NGS.

## Case presentation

### Clinical information

The proband was a male infant, uneventful cesarean delivery by a 33-year-old G2P2 mother. He was born to nonconsanguineous healthy parents without the family history of metabolic diseases. The patient was the second child of the family, his 10-year-old sister was healthy. He was born at 37 + 6 weeks of gestation with a birth weight of 3500 g and Apgar Score of 10 at 5 min. No obvious malformations were noted at birth. On the 5th day of age, he was referred to the local hospital with a 4-h history of shortness of breath, poor reaction, lethargy, and poor crying. After the examination, he was diagnosed with neonatal pneumonia, hypoxic-ischemic encephalopathy (HIE), and neonatal hyperbilirubinemia. He was treated supportively and appeared to improve the clinical manifestations. Subsequently, he was discharged home on the 14th hospital day.

At the age of 26 days, he was transferred to the Wuhan Medical & Health Center for Women and Children with a history of poor reaction and decreased feeding since birth. The tests of blood gas analysis, routine blood test, plasmic electrolyte, myocardial enzymogram, cerebrospinal fluid, and liver function were performed. The results indicated increased creatine kinase isoenzyme MB (CK-MB) level of 116 U/L (normal range 0–24 U/L), increased creatine kinase CK level of 221 U/L (normal range 30–170 U/L), decreased platelets (PLT) level of 25 × 10^9^/L (normal range 242–378 × 10^9^/L) (Table S1).

MS/MS (UPLC-TQD, WATERS, USA) showed significantly elevated propionyl carnitine level of 10.111 uM (normal range 0.5–5 uM), and propionyl carnitine/acetyl carnitine ratio at 1.373 (normal range 0.04–0.25), which was indicative of methylmalonic acidemia (MMA) or PA. Urine organic acid analysis with GC/MS showed elevated 3-hydroxypropionate (normal range 0–1.1 (u)mol/mol creatinine), propionyl glycine (normal range 0 (u)mol/mol creatinine), and methylmalonate (the normal range 0.2–3.6 (u)mol/mol creatinine), which indicated PA (Table 1). Serum total homocysteine was 20.16 umol/L (normal range 5–15 umol/L). Serum VitB12 was 184.5 pmol/L (normal range 100–300 pmol/L), folic acid was 17.68 nmol/L (normal range 11-54 nmol/L). The brain CT demonstrated that the density of falx cerebri raised slightly, diffuse symmetry density of the white matter of bilateral brain parenchyma decreased, with unshifted midline structure. Therefore, the proband was diagnosed as mild neonatal hypoxic-ischemic encephalopathy (HIE). Brain ultrasound showed no obvious abnormalities. Electroencephalogram (EEG) showed a little more multifocal spike discharges, especially in the bilateral frontal region. The brain magnetic resonance imaging (MRI) performed at the age of 40 days demonstrated decreased T1/T2, FLAIR and DWI signals in the white matter of bilateral brain parenchyma, slightly wider in outer space of the frontotemporal region. Based on these results, the infant was clinically characterized as PA. He was treated with antibiotics treatment, phenobarbital, L-carnitine, folic acid, hydroxycobalamin, and protein restriction. GC/MS testing at the age of 39 days showed that the results were improved than previous tests (Table 1).

**Table 1:** The results of MS/MS and GC/MS during the proband’s hospitalization

Age	MS/MS		GC/MS			
	C3 (0.5–5 μM)	C3/C2 (0.04–0.25)	Methylmalonate (0.2–3.6 (u)mol/mol creatinine)	3-Hydroxypropionate (0–1.1 (u)mol/mol creatinine)	Propionyl glycine (0 (u)mol/mol creatinine)	Methyl citrate (0–1.1 (u)mol/mol creatinine)
27 days	10.111	1.373	0.86	1654.59	162.68	196.82
39 days	/	/	0.00	9.34	0.00	28.38

C3: propionyl carnitine, C2: acetyl carnitine, “/”: unknown

At the age of 45 days, he was hospitalized again. After admission, the patient’s condition continued to worsen, he developed a seizure, lethargy, irritability. Subsequently, he was referred to intensive care unit (ICU) for therapy. Finally, his family withdrew aggressive therapies after 6 days treatment and he was discharged from the hospital. The outcome was poor, the patient was dead at the age of nearly 2 months.

### Targeted next-generation sequencing

In order to identify the etiology, targeted NGS was performed on peripheral blood DNA samples from the patient. The captured DNA array was described in the previous study [17]. The captured DNA probes of the *PCCA* and *PCCB* target regions were listed in Table S2. The target region with 3867 bp of the *PCCA* and *PCCB* gene was for the subsequent analysis. The DNA sample of the case was fragmented to generate 200–250 bp paired-end library by Covaris LE220 (Massachusetts, USA). Library Construction was conducted as a previously published procedure [18]. The final amplified library was sequenced on the BGISEQ-500 platform (Shenzhen, BGI) to generate 90 bp paired-end reads. The original sequencing data were analyzed using previously published criteria [18].

The average coverage is 100.00% of the target region, and the average sequencing depth is 108.26 fold. Targeted NGS identified only one heterozygous mutation c.937C > T (p.Arg313Ter) (NM_000282) on exon 12, and no CNV detected on exon-level. Since the clinical phenotype of the proband was consistent with the PA patient, and PA is an autosomal recessive disease, we checked the sequencing reads of the whole genome of the *PCCA* gene in the binary alignment map (BMA) file and noticed some reads were unable to match the reference. This inspired us to find deletion inside the exon. Therefore, we retrieved these reads and found a heterozygous mutation chr13:100915039-100915132delinsAA.It named as c.773_819 + 47delinsAA (GRCh37.p13) according to Mutalyzer (https://mutalyzer.nl/position-converter, released on 11 June 2018). It is a novel complex deletion–insertion, which results in a 94 bp deletion encompassing the last 47 nucleotides of exon 10 plus the first 47 intron bases and 2 bp AA insertion of the *PCCA* gene.

### Confirmation of the candidate mutations in the family

**Fig. 1:**
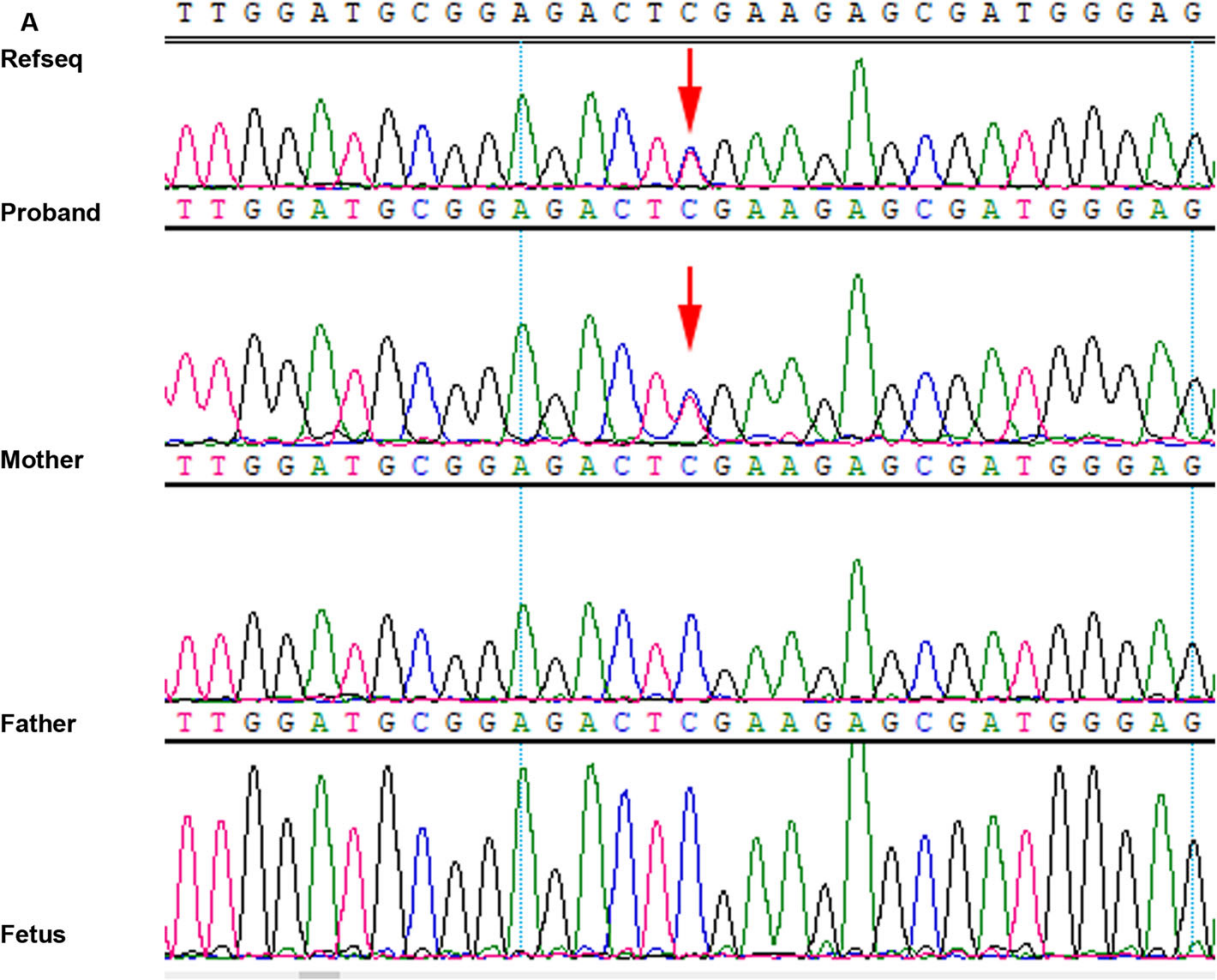
The nonsense mutation detected in the family using Sanger sequencing. The proband and his mother are heterozygous, while the father and the fetus are the same as the reference sequence. Arrow indicates mutation site

**Fig. 2:**
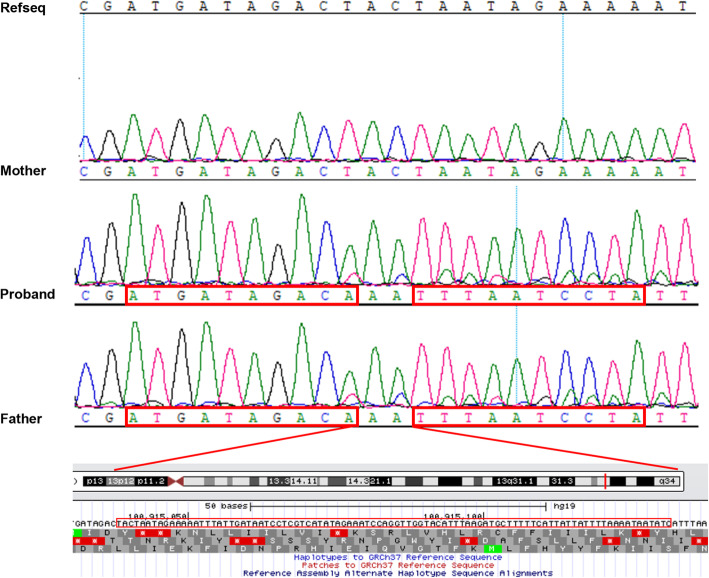
The delins c.773_819 + 47delinsAA mutation detected in the family using Sanger sequencing. The proband and his father are heterozygous, while the mother is the same as the reference sequence. The red box shows the breakpoint location

Sanger sequencing and quantitative PCR (qPCR) were performed to confirm the existence of the identified variant in the patient and his parents, respectively. The pairs primers for c.937C > T mutation were F-TCTCCCAGTTTTTGGCTCGAA, R-AAGGGTGGAGAAGGAAGGGT. The pairs primers for c.773_819 + 47delinsAA mutation were F-CTTCTCCTTCTTCCCCTTCC, R-AGCACAGGAGTCCAGGTCAG. The mutation c.937C > T was detected in the proband and his mother. (Fig. 1). The mutation c.773_819 + 47delinsAA was detected in the proband and his father (Fig. 2). The deletion sequence is TACTAATAGAAAAATTTATTGATAATCCTCGTCATATAGAAATCCAGGTTGGTACATTTAAGATGCTTTTTCATTATTATTTTAAAATAATATC (Fig. 2).

**Fig. 3:**
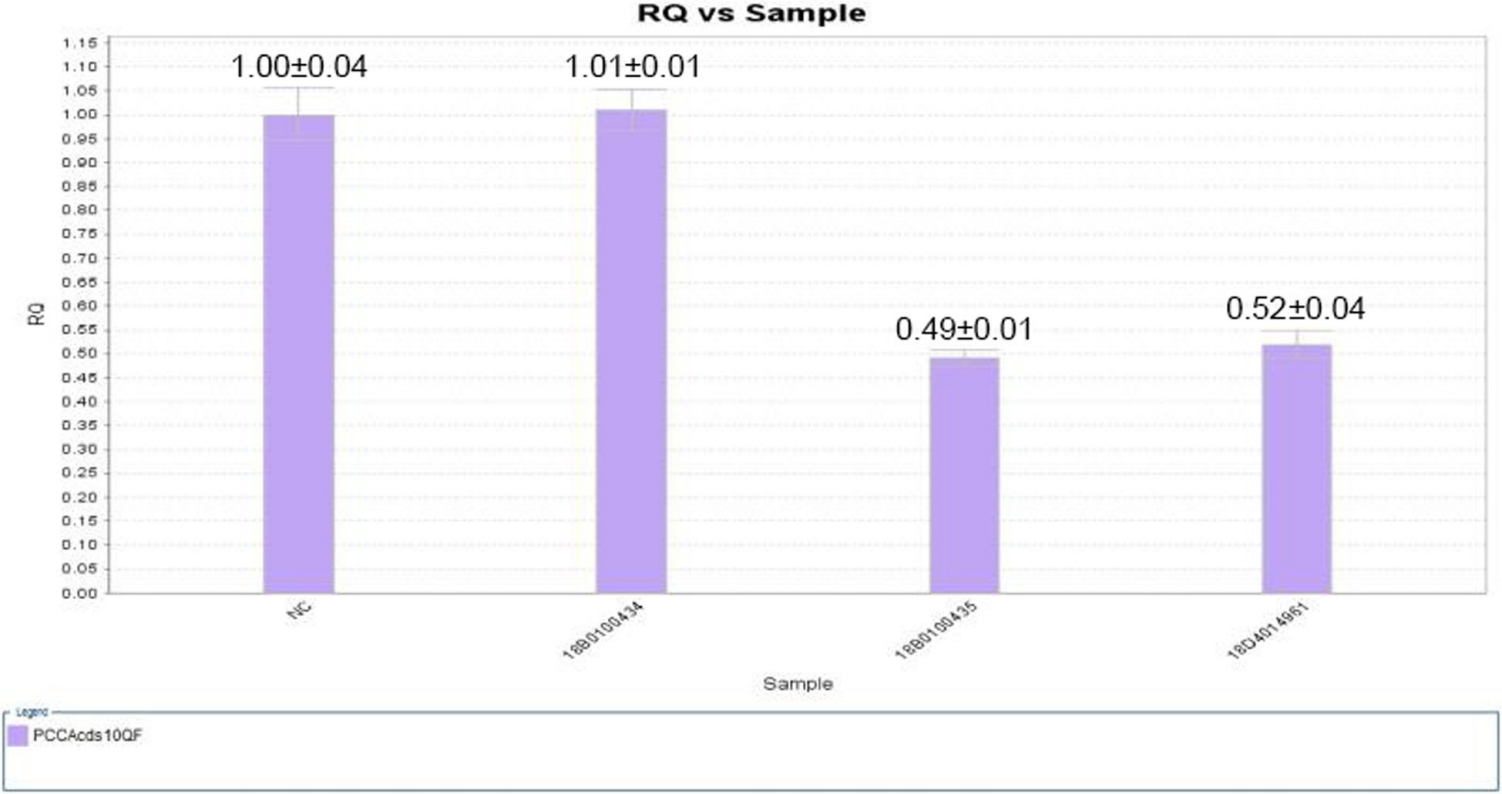
Confirmation of the delins mutation using qPCR. Relative quantification (RQ) of the DNA copy number of the partial exon 10 (Purple pillars) was selected for an independent determination. NC refers to the control sample, 18B0100434 refers to the mother of the patient, 18B0100435 represents the father of the patient, 18D4014961 refers to the patient. The error bars represent standard deviation (SD) of the predicted value. Numbers shown are mean values ± SD of each sample. RQ = 2^-△△Ct^

Quantitative PCR (qPCR) was used to detect the DNA copy number of the partial exon 10 in the proband and his parents, with one primer designed to hybridize the deletion region, the other one for the upstream or downstream of the deletion region. The primers are as follows, F-GCTGCTTCTAGTTTTGGCGA, R-ACCAACCTGGATTTCTATATGACGA. The results showed that the quantity of partial exon 10 in the DNA sample of the proband was close to the father, almost 50% of his mother and the control sample (Fig. 3).

The results above indicated that the proband’s healthy parents were heterozygous carriers. The heterozygous c.937C > T allele was inherited from his mother. The c.773_819 + 47delinsAA allele was inherited from his father.

### Prenatal diagnosis and newborn screening

The parents were willing to have a healthy child and expressed a desire to undergo prenatal diagnosis in the subsequent pregnancy. We collected 20 ml amniotic fluid samples approximately at 18-week gestation from the mother under ultrasound guidance. Extraction of fetal DNA from the amniotic fluid was performed using the QIAamp DNA Mini Kit (Qiagen, Hilden, Germany). Sanger sequencing was performed to validate the status of the mutations in the fetus. The pairs primers of the mutations were the same as that of validation in the family. Meanwhile, elimination of maternal contamination was proceeded according to a paper reported [19]. The result showed the fetus carried neither of the two mutations. Therefore, the couple decided to continue the pregnancy and subsequently gave birth to a healthy infant. Newborn screening of inherited metabolic diseases (including PA) with MS/MS (UPLC-TQD, WATERS, USA) was carried out, the result was normal.

## Discussion and conclusion

PCC enzyme is located on the mitochondria and it functions in catalysis of the ATP-dependent carboxylation of propionyl-CoA to D-methylmalonyl-CoA. PCC enzyme is composed of α and β subunits. The α subunit contains the biotin carboxylase (BC), as well as the biotin transfer (BT) and biotin carboxyl carrier protein (BCCP) domains. BC domain is responsible for catalyzing the MgATP-dependent carboxylation. It consists of A, B, C subdomain [9, 20, 21]. The α subunit is encoded by the *PCCA* gene. To date, 102 mutations have been described in the *PCCA* in Human Gene Mutation Database (http://www.hgmd.cf.ac.uk/ac/index.php, professional 2019.4). The mutation spectrum of the *PCCA* gene was highly heterogeneous without predominant mutations, whereas a limited number of mutations are highly frequent in the *PCCB* gene [22]. In the whole, among the types of mutations reported in the *PCCA* gene, the missense mutations are predominant of the genetic defects in *PCCA* gene, followed by small indels and splicing mutations. It was also reported that a high frequency of genomic deletions affecting the *PCCA* gene in PA patients [23].

In our report, we identified the compound heterozygous mutations in the patient.

The mutation p.Arg313Ter originated from proband’s mother. It is the most frequent pathogenetic mutation in the *PCCA* gene previously reported [24, 25]. The recurrence of this mutation ascribed to influence the hypermutable CpG dinucleotides [26]. The other mutation is c.773_819 + 47delinsAA. It is a novel complex deletion–insertion (delins) mutation which originated from proband’s father. It encompasses the last 46 bp coding region of the exon 10 and the first 47 bp of the intron 10. As the length of contiguous nucleotide deletion in exon 10 is not multiples of 3 bp. The partial deleted sequence in exon 10 with 46 bp may shift the translational reading frame. The 47 bp deletion in intron 10 may affect the donor splice site of exon 10. It may disrupt exon 10 splicing as the abolition of the 5′ splice site and result in an unstable protein structure theoretically. The delins mutation in this report may influence the C subdomain of the BC domain of PCC. Therefore, it was predicted to affect the catalysis or substrate binding. This mutation has not been presented in the HGMD database or reported previously. Therefore, it suggests that it is a novel pathogenic variant. The compound mutations identified here may have a severe impact on the protein function, resulting in a severe phenotype of PA. However, the consequence of this delins mutation was not determined by transcript analysis due to there were only DNA samples obtained in this study.

The delins is a complex lesion that appears to represent a combination of deletion-insertion. It is a relatively rare type of mutation resulting in human genetic disorder and has been described in many different genes [27]. To our knowledge, several delins mutations have been reported in the *PCCB* gene, one mutation c.1218_1231del14ins12 is frequently found in 32% of Caucasian population [2, 28]. Another delins mutation c.101_105 + 5delinsGCAACGGG has been reported in PC*CA* with several PA recently [29]. For this reason, the complex c.773_819 + 47delinsAA is the second delins reported in the *PCCA* gene.

There was no straightforward genotype–phenotype correlation in PA deficiency since clinical manifestations in PA patients were heterogeneous. As previous reports showed that the majority of patients bearing null variants in both alleles were associated with the most severe phenotype with early-onset [30]. In our case, the patient possessed two null mutations in alleles. Additionally, the clinical course of the patient was severe, with neonatal-onset, along with the demise at the age of around 2 months. It was concordant with the above genotype–phenotype correlation rule. Taken together, our result broadened the genotype–phenotype correlation of the *PCCA* gene in PA patient.

NGS has strong ability to detect point mutations and CNV in exon level. However, it is poor at identifying the deletion/ repetition inside the exon. In this case, the clinical phenotype of the proband was very consistent with the PA patient and a highly relevant pathogenic mutation has been detected. In cases like this, it is necessary to manually check the BAM diagram. There will be some interesting findings. The detection rate of NGS can be improved when combined with other verification methods. It is useful for the cases that the NGS result revealed only a heterozygous mutation in autosomal recessive disease when the gene is associated with phenotypes.

In summary, we report a Chinese patient with severe early-onset PA syndrome resulting from a recurrent nonsense c.937C > T and a novel delins c.773_819 + 47delinsAA mutations in *PCCA* gene using the targeted NGS. This is the second report of PA patient with delins mutation in *PCCA* gene. Our result broadens the mutation spectrum of the gene *PCCA*.

Targeted NGS is a valuable and cost-effective method to illustrate the precise comprehensive diagnosis of the hereditary disorder. For cases that the NGS results revealed only a heterozygous mutation in autosomal recessive disease when the gene is associated with phenotypes, it is necessary to manually check the BAM diagram. Molecular diagnosis is an effective way to assist in genetic counselling and prenatal diagnosis for family members.

## Supplementary information

**Additional file 1: Table S1.** Laboratory findings of the proband.

**Additional file 2: Table S2.** The captured DNA probes of the *PCCA* and *PCCB* target region.

## Data Availability

The data generated in this study have been deposited into CNGB Sequence Archive (CNSA: https://db.cngb.org/cnsa; accession number CNP0000185).

## Abbreviations

BAM: Binary alignment map
BC: Biotin carboxylase
BCCP: Biotin carboxyl carrier protein
BT: Biotin transfer
EEG: Electroencephalogram
GC/MS: Gas chromatography-mass spectrometry
HIE: Hypoxic-ischemic encephalopathy
ICU: Intensive care unit
MMA: Methylmalonic acidemia
MRI: Magnetic resonance imaging
MS/MS: Mass spectrometry
NGS: Next-generation sequencing
PA: Propionic acidemia
PCC: Propionyl-CoA carboxylase
qPCR: Quantitative PCR
