# Supplementary material for: A novel delins (c.773_819+47delinsAA) mutation of the PCCA gene associated with neonatal-onset propionic acidemia: a case report
Source: BMC Med Genet. 2020 Aug 20;21:166. doi: 10.1186/s12881-020-01102-1 (PMC7441651; doi:10.1186/s12881-020-01102-1)
Supplement: Supplementary file 1 — Additional file 1: Table S1. Laboratory findings of the proband. [file 12881_2020_1102_MOESM1_ESM.docx]

Table S1. Laboratory findings of the proband.

| **Characteristics** | **26 days** | **39 days** | **47 days** | **Units** | **Reference range** |
| --- | --- | --- | --- | --- | --- |
| pH | 7.12 | 7.54 | 7.44 |  | 7.35-7.45 |
| pCO2 | 17.6 | 28 | 25 | mmHg | 35-45 |
| pO2 | 94 | 63 | 85 | mmHg | 75-110 |
| sO2 | 94.4 | 94 | 97 | % | 95-98 |
| SBE | -22.3 | 1 | -7 | mmol/L | -3-3 |
| HCO3- | 5.5 | 24 | 16 | mmol/L | 21.4-27.5 |
| HCT | 29.2 | 41.6 | / | % | 33-42 |
| PLT | 25 | / | 428 | 10^9^/L | 100-578 |
| WBC | 4.81 | 10.15 | 5.8 | 10^9^/L | 5-12 |
| RBC | 2.95 | 4.3 | 3.84 | 10^12^/L | 3.2-4.9 |
| CK-MB | 116 | 114 | 60 | U/L | 0-24 |
| CK | 221 | / | / | U/L | 30-170 |
| hs-CRP | 7.28 | 4.41 | / | mg/L | 0-3 |

pCO2 = partial pressure of carbon dioxide, pO2 = partial pressure of oxygen, sO2 = oxygen saturation, SBE = Standard Base Excess, HCO3- = Actual Bicarbonate Radical, HCT=hematocrit, PLT = platelet, WBC = white blood cell, RBC = red blood cell, CK =creatine kinase, CK-MB=creatine kinase-MB, hs-CRP = high-sensitivity C-reactive protein
